# Enhancing Medical Committee Decision-Making: Insights from Qatar

**DOI:** 10.5339/qmj.2026.26

**Published:** 2026-06-10

**Authors:** Sabrina Arrouf, Abdelbasset Ghalgaoui, Saad Alkaabi, Yagoub Alhamadi, Nawal Altamimi, Khalifa Almansouri, Rajvir Singh, Moza Alishaq, Mohamad Hassan Elkordy, Maysa Abdelmageed, Tintu Mathew, Rasha Abdelbaky, Betsy Varughese, Asrar Albaz, Wael Gaad, Mohamed Qaddoura, Zahra Makki, Amr Elshorkobaly, Shemmy Gutay, Nihal Gabora, Maryam Ali, Sheila Ambrosio, Ana Antonio, Mustafa Butt, Esmail Maik, Noufal Kunniyullathil, Jala Mohamed, Saad Shahbal, Abdulmutaleb Alahmar, Shaikha Alkorbi, Alhareth Aljabor, Reem AlHadad, Nasser Gaasha, Viswapriya Pillai, Monisha Kurup, Sherlane Melo

**Affiliations:** 1 International Medical Affairs Office (IMAO), Hamad Medical Corporation (HMC), Doha, Qatar; 2 General Medicine Practice, Val-de-Marne, France; 3 Department of Nursing, Hamad Medical Corporation (HMC), Doha, Qatar; 4 Institut Universitaire de Formation des Cadres (INUFOCAD), Port-au-Prince, Haiti; 5 Gastroenterology, Medicine Department, Hamad Medical Corporation (HMC), Doha, Qatar; 6 Administration Medical Education, Heart Hospital, Hamad Medical Corporation (HMC), Doha, Qatar; 7 Corporate Quality Improvement, Quality and Patient Safety, Hamad Medical Corporation (HMC), Doha, Qatar; 8 Al Wakra Hospital, Hamad Medical Corporation (HMC), Doha, Qatar; 9 Accident and Emergency Department, Hamad Medical Corporation (HMC), Doha, Qatar; 10 Department of Research, Hamad General Hospital, Hamad Medical Corporation (HMC), Doha, Qatar; 11 Department of Medicine, Hamad Medical Corporation (HMC), Doha, Qatar; 12 Corporate Cancer Services, National Center for Cancer Care and Research (NCCCR), Hamad Medical Corporation (HMC), Doha, Qatar

**Keywords:** Cross-Border Healthcare, Clinical Decision-Making, Healthcare Committees, Health Policy, Quality Improvement, Patient-Centered Care

## Abstract

**Background:**

Treatment Abroad (TA) programs are a key component of healthcare delivery for complex cases requiring highly specialized care. In Qatar, the International Medical Affairs Office (IMAO) manages over 11,000 TA requests annually through multidisciplinary committees and specialized subcommittees to support evidence-based decision-making. Despite advances in committee structures and the adoption of hybrid meeting models, challenges persist in optimizing efficiency, transparency, and committee member satisfaction, highlighting the need for systematic evaluation of committee processes and composition.

**Objectives:**

To identify challenges affecting decision-making and operational effectiveness within IMAO Treatment Abroad committees, define characteristics of optimal committee composition, and develop a reproducible framework to enhance transparency, communication, and committee practices across the IMAO and similar healthcare organizations.

**Methodology:**

Between July and December 2024, a cross-sectional survey was conducted among 162 CMs from main and specialized subcommittees using a home-developed instrument combining Likert-scale, yes/no, and open-ended questions; the tool was pilot-tested for reliability (Cronbach’s α = 0.82). Quantitative data were analyzed using non-parametric tests, while qualitative data underwent dual-coded thematic analysis.

**Results:**

A total of 87/162 committee members (CMs) responded (53.7%), predominantly male (72.4%) and highly experienced within HMC (≥9 years, 86.2%), while 50.6% had similar experience within the IMAO committee. Most preferred remote or hybrid meetings (67.8%) and email communication (51.7%), with high satisfaction reported for workflows (93.1%) and IMAO collaboration (65–69%). While 81.6% reported no external influence on decisions, 23% identified the absence of subcommittee input as a barrier, and 52.9% supported anonymized patient requests to enhance transparency. Greater organizational experience was associated with higher satisfaction and increased perception of subcommittee absence as a barrier (36.4% vs. 9.3%; p < 0.05), whereas committee-specific experience showed no significant effect. Key challenges included communication gaps with overseas facilities, interface usability, incomplete medical information, lack of financial compensation (95.4%), and exposure to patient reprisals.

**Conclusion:**

Medical committee effectiveness in Treatment Abroad programs is influenced by committee members’ organizational experience and perceptions of decision-making processes. Findings underscore the need for structured multidisciplinary input, transparent governance, streamlined communication, and diverse committee representation across gender and experience levels to support equitable and consistent decision-making. The proposed hierarchical framework provides a practical model to standardize processes, enhance transparency, and optimize committee performance within IMAO and comparable healthcare settings.

## 1. INTRODUCTION

Treatment Abroad (TA) programs represent a substantial component of healthcare delivery for complex and highly specialized cases, requiring timely, transparent, and well-coordinated decision-making processes. The International Medical Affairs Office (IMAO)^1^ in Qatar manages a consistently high volume of TA requests, exceeding 11,000 annually. Each request requires careful review, coordination among stakeholders, and support for committees’ members (CMs) in making informed decisions based primarily on the availability and feasibility of treatment within national healthcare facilities as well as international treatment options.

Over the past decade, the IMAO has developed 27 specialized subcommittees across Hamad Medical Corporation (HMC)^2^ and Sidra Medicine,^3^ representing a broad range of clinical specialties, including but not limited to pediatrics, cardiology, transplant medicine, and plastic surgery. These subcommittees provide expert input on complex cases, advising the main IMAO committee to ensure structured, evidence-based decision-making, while the IMAO main committees retain final decision-making to ensure transparency and minimize potential bias.

The transition to remote and hybrid meeting formats, accelerated by the COVID-19 pandemic,^4^ has further improved communication efficiency and operational flexibility. Despite these structural advances, IMAO TA committees continue to face challenges related to operational efficiency, committee members (CMs) satisfaction, and maintaining transparent decision-making processes. The IMAO committees’ coordinators have frequently received informal feedback from CMs regarding committee processes and decision-making challenges. Building on these insights, and to capture CMs’ experiences more systematically, we conducted a survey to generate quantifiable data. The survey was also motivated by a reflection regarding optimal committee compositions, whether committee membership should be rotated or maintained over longer duration. Further consideration was given to defining evidence-based criteria for committee membership, including the attributes of an “ideal” committee member. The findings aim to support data-driven improvements in committee composition, efficiency, transparency, member satisfaction, governance practices and ultimately patient outcomes.

### 1.1 Study objectives

To identify challenges affecting medical committee decision-making processes and operational effectiveness within the IMAO Treatment Abroad committees.

To identify key characteristics of optimal committee composition, including the attributes of an ideal committee member and considerations related to member continuity versus rotation.

To develop a reproducible framework, informed by IMAO’s experience, aimed at enhancing transparency, communication, and committee practices, with potential applicability across similar healthcare organizations.

## 2. METHODS

### 2.1 Study design and participants

A cross-sectional study was conducted between July 30 and December 10, 2024, to assess CMs’ experiences and perceptions of TA decision-making processes. The study population included 162 CMs from the main committees and various specialized subcommittees. The survey was developed collaboratively by the IMAO physician coordinators and experts in medical affairs and survey methodology, ensuring that it captured both generalizable aspects of healthcare committee operations and specific contextual insights from IMAO as an illustrative case.

This study constitutes the initial phase of a multi-phase quality improvement initiative aimed at improving the decision-making process of the IMAO TA committee. Data collection via survey was conducted to assess current committee functioning, thereby establishing a reference point against which future changes and interventions can be evaluated. Subsequent phases will involve implementation of a structured decision-making framework based on these findings, followed by reassessment to measure potential impacts and improvements in committee satisfaction, transparency, and operational effectiveness.

### 2.2 Survey instrument

The survey combined quantitative and qualitative components. Quantitative items included Likert-scale and dichotomous (yes/no) questions, while open-ended questions captured qualitative insights. The instrument assessed participant characteristics, committee organization, communication preferences, decision-making challenges, and perceptions of committee effectiveness. The survey was distributed electronically via Google Forms, with participation being anonymous and voluntary. To improve response rates, reminder emails and follow-up phone calls were conducted throughout the data collection period. The complete survey instrument is provided in Appendix 1.

### 2.3 Ethical considerations

This quality improvement project was conducted in accordance with the ethical principles outlined in the Declaration of Helsinki. Ethical approval was obtained from the Institutional Review Board (IRB) at HMC (approval no: 0109-2024). Participants were informed of the voluntary nature of their involvement and assured of confidentiality and anonymity. Informed consent to participate was obtained from all participants before their involvement in the project. Data management and oversight were conducted by the Corporate Quality and Patient Safety Department at HMC to ensure data integrity and privacy. According to the policies of our institution, quality improvement projects that do not constitute human-subjects research do not require formal IRB approval.

### 2.4 Validity and reliability

To ensure content validity, the survey underwent expert review by professionals in medical affairs, committee operations, and survey methodology. Face validity was confirmed through feedback from subject matter experts and a pilot group of ten participants (*n* = 10). The instrument was refined over two rounds of pilot testing to improve clarity, structure, and relevance. Test-retest reliability was assessed by administering the survey to the same participants 15 to 18 days apart. Internal consistency of the quantitative items was evaluated using Cronbach’s alpha (α = 0.82), indicating strong reliability.

### 2.5 Data analysis

Quantitative data were analyzed using the Statistical Package for the Social Sciences (SPSS), version 26. Normality testing demonstrated a deviation from normal distribution (p < 0.05); therefore, non-parametric methods were used for analysis. Comparisons between groups (<9 years vs. ≥9 years of experience) were performed using Mann–Whitney U tests. Associations between categorical variables were examined using Chi-square or Fisher’s Exact tests. Qualitative data from open-ended survey responses were analyzed using thematic analysis. Two researchers independently coded responses to ensure consistency and reduce bias. Discrepancies were resolved through discussion to reach consensus, and the coding scheme was refined iteratively to identify recurrent themes. Representative quotes were included for each major theme to illustrate how participant responses supported the identified patterns. This approach provides both quantitative rigor and qualitative depth, allowing for a reproducible framework that can be adapted to other healthcare committee settings.

## 3. RESULTS

The key insights and trends from survey results were summarized in Table 1. A total of 87 out of 162 eligible CMs completed the survey, corresponding to a 53.7% response rate. Respondents were predominantly experienced, with 86.2% reporting ≥9 years of service at HMC, and 50.6% having ≥9 years of involvement with the IMAO committee. The study was conducted in 2024, and the cutoff of 9 years was used to distinguish members who joined IMAO close to its establishment in 2013 from more recent members. The sample was composed mostly of male participants (72.4%).

Regarding meeting and communication preferences, 67.8% of respondents preferred remote or hybrid formats, while 32.2% favored face-to-face meetings. Over half (51.7%) indicated a preference for email as the primary communication channel. Satisfaction with the existing workflow was high, with 93.1% expressing satisfaction with the weekly meeting schedule, and 55.2% reporting that they routinely allocate additional time to support TA committee responsibilities.

CMs’ perception of their interactions with the IMAO team members were generally positive. Specifically, 65% of respondents were satisfied with collaboration with the IMAO team, and 68.9% expressed satisfaction with the support provided by the IMAO physician coordinators. With respect to decision-making processes, 23% perceived the lack of sub-committee recommendations as a barrier. More than half (52.9%) reported that anonymizing patient requests would improve transparency and facilitate more objective decision-making compared with the current process in which patient identities are disclosed. Furthermore, 81.6% stated that their decisions were not influenced by external actors.

Feedback regarding system usability indicated that 18.6% experienced challenges with the online IMAO interface. Finally, the majority of the participants (95.4%) did not receive financial compensation for their role on the TA committee.

Table 2a shows that employees with ≥9years of experience within the corporation (HMC) rated all three aspects of IMAO services more positively than those with less experience. Significant differences were observed for the relationship with the IMAO coordinators (*P* = 0.024), satisfaction with the medical summary (*P* = 0.011), and the help provided by the IMAO team (*P* = 0.035). This suggests that longer-tenured employees tend to have a more favorable perception of their interactions with IMAO coordinators, the quality of the medical summary, and the support provided by the IMAO team.

In contrast toTable 2a, where committee members’ length of experience within the corporation (HMC) tended to significantly influence overall satisfaction with the Treatment Abroad Committee processes handled by the IMAO team, Table 2b demonstrates that the length of service within the IMAO committee itself did not have a significant impact on committee members’ perceptions. This suggests that organizational tenure, rather than committee-specific experience, plays a more influential role in shaping satisfaction with the IMAO team and its services.

**Figure 1.**
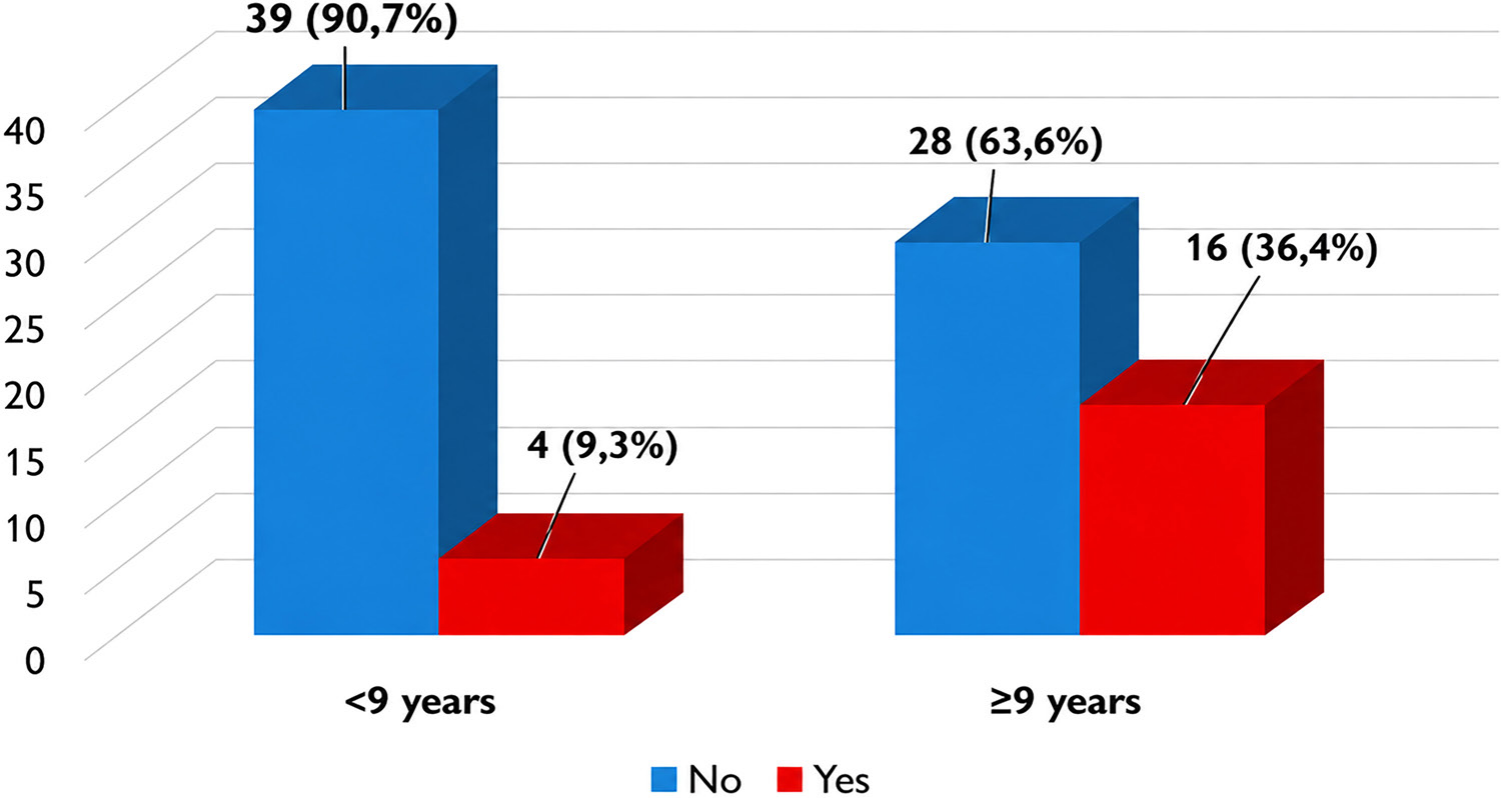
Perception of absence of sub-committee decisions as an obstacle in decision-making by experience level at HMC (*N* = 87).

Figure 1 shows the perceptions of the absence of sub-committee decisions as an obstacle in decision-making by experience level. Among participants with <9 years of experience, : the majority (90.7%) did not perceive it as an obstacle, while only a small proportion (9.3%) did. In contrast, among those with 9 years of experience or more, a notable proportion (% 36.4) considered the absence of sub-committee decisions an obstacle. This difference was statistically significant (*P* = 0.003).

Table 3 represents the qualitative feedback from TA CMs, highlighting several key areas for improvement. Participants emphasized the need to enhance the digital interface, suggesting the introduction of tutorials, Frequently Asked Questions (FAQs), fewer login steps, and a mobile application to facilitate easier access. Improvements in communication were also recommended, including clearer decision rationales, more specific questions for members, and additional feedback mechanisms. Concerns were raised regarding the completeness and relevance of medical information from overseas facilities, with members noting that gaps or irrelevant data could affect both decision accuracy and post-approval follow-up care. Several respondents expressed frustration over the lack of financial compensation for additional workload, particularly for tasks completed outside clinical hours, and stressed the importance of transparency, including the publication of annual statistics and conflict-of-interest (COI) declarations. Participants also highlighted the need for patient education to encourage trust in local treatment options and reported concerns about personal safety when dealing with aggressive applicants. Despite these challenges, feedback regarding the support and responsiveness of the IMAO team was consistently positive, reflecting strong collaboration and assistance in managing the TA process.

## 4. DISCUSSION

### 4.1 Gender representation within the IMAO committees

Of the 162 invited IMAO CMs, 87 responded to the survey (53.7%). Among respondents, 24 (27.6%) were female and 63 (72.4%) were male. The proportion of female respondents (27.6%) was comparable to the proportion of women among invited committee members (24.1%), indicating a gender imbalance within the committee. This proportion closely mirrors global findings: the World Health Organization reports^5^ that women represent roughly 70% of the healthcare workforce but hold only about 25% of leadership positions, underscoring a persistent gap. Similarly, Gopal et al.^6^ found that women’s representation in senior healthcare leadership often remains below 30%, a figure comparable to our committee’s 27.6%, and attributed this disparity to implicit and unconscious biases in leadership selection. Al-Thani^7^ highlighted that in Qatar, women’s leadership opportunities remain constrained by institutional obstacles, patriarchal norms, and limited access to mentorship, despite formal commitments to gender equality. Taken together, these findings suggest that both implicit cognitive biases and systemic barriers contribute to gender underrepresentation in leadership.

### 4.2 Committee length of service versus organizational familiarity

Kugel and Mercado^8^ emphasize that member satisfaction and self-reported perceptions are important indicators of committee and board effectiveness, particularly within nonprofit and healthcare organizations. In line with this framework, the present findings suggest that institutional experience, rather than prolonged tenure on a specific committee, plays a more substantial role in shaping member satisfaction and perceived committee functioning.

The present findings challenge the assumption that prolonged tenure on a committee is a primary determinant of member satisfaction or committee effectiveness. Committee members with shorter tenure reported satisfaction levels comparable to those of long-serving members, indicating that duration of committee service alone does not significantly influence perceived committee functioning. Instead, satisfaction was more strongly associated with broader organizational familiarity. Members with greater institutional experience demonstrated higher satisfaction regardless of the duration of their committee service.

These findings suggest that organizational knowledge, contextual understanding, and familiarity with internal processes may contribute more meaningfully to effective committee participation than extended tenure on a single committee. Members with greater institutional familiarity may be better equipped to understand organizational priorities, navigate administrative structures, and contribute more confidently to committee discussions and deliberations.

The findings also have important implications for committee composition and continuity. Since satisfaction and perceived effectiveness appear to be driven more by institutional knowledge than by prolonged committee tenure, committee membership may be refreshed or rotated without negatively affecting committee functioning, provided that members possess sufficient organizational experience. This supports the notion that strategic rotation of committee members can maintain effectiveness while also promoting diversity of perspectives and reducing the potential limitations associated with excessive tenure.

### 4.3 Meeting preferences and workload

A total of 67.8% of CMs preferred remote or hybrid meetings. This finding mirrors Chen et al.’s^9^ study of a heart transplant committee, which showed healthcare professionals favored remote meeting formats due to increased flexibility, better work-life balance, and improved accessibility. Chen et al. found that virtual and hybrid meetings allowed members to manage clinical and administrative duties more efficiently and promoted greater participation across dispersed teams. Although 93.1% of respondents in our study were satisfied with the weekly meeting schedule, 55.2% reported working extra hours, echoing Sjetne et al.’s^10^ findings that physicians with non-clinical responsibilities face heavier workloads unless dedicated time is allocated for these duties.

### 4.4 Transparency and gendered perceptions of anonymous patient requests

Over half (52.9%) of participants believed anonymous patient requests could improve decision-making transparency. Experience level did not significantly affect this belief (*P* = 0.91), but a gender difference was observed (*P* = 0.02), with 60.3% of male participants supporting anonymity, compared to fewer female participants. Female members tended to view anonymity as less critical, expressing confidence that their decisions would remain consistent regardless of patient anonymity. This aligns with findings from Eagly and Karau’s^11^ meta-analysis, which highlighted that female leaders’ style is often characterized by fairness, collaboration, and ethical standards, contributing to equitable decision-making processes. This may explain the confidence of female members in the committee’s impartiality despite the structural gender imbalance.

Both genders largely agreed that third-party recommendations did not influence decisions, indicating that susceptibility to external pressures is not gender dependent.

### 4.5 Experience level and perception of the subcommittee in decision-making

A significant association was observed between years of experience and the perception of subcommittee opinion as essential to the decision-making process (*P* = 0.003; Figure 1). Only 9.3% of participants with <9 years of experience considered the absence of subcommittee input an obstacle, compared to 36.4% among those with ≥9 years. This suggests that more experienced CMs value subcommittee recommendations in making confident decisions on whether a patient deserves TA or can be managed locally.

This pattern aligns with the Dunning-Kruger effect, a cognitive bias in which individuals with limited experience may overestimate their understanding or competence, while those with greater expertise are more likely to recognize system gaps and limitations.^12^ In this context, junior CMs may underestimate the role of subcommittees due to limited exposure to nuanced or borderline cases where specialized input is critical. Conversely, senior members, having navigated such complexities, are more sensitive to how missing subcommittee evaluations can affect decision clarity and quality. These findings underscore the importance of orientation and mentorship for newer members.

### 4.6 Awareness of bias and influence of external factors

Interestingly, 100% of less experienced members felt that family names did not influence decisions, compared to 86.4% of more experienced members, suggesting greater awareness of subtle biases among senior CMs. This aligns with a study published in the Journal of General Internal Medicine, which found that as professionals gain experience, they become more attuned to non-clinical factors that might impact decisions, such as family background and social influences.^13^

### 4.7 Challenges with non-medical requests and committee structure

Non-medical requests, such as sponsorship of a patient’s escort, who may be a family member or not, and airplane ticket class (e.g., business when required for medical reasons), were considered challenging by 56.3% of participants. This highlights the need for clear criteria and possibly a dedicated subcommittee, aligning with Brown et al.^14^, who found that specialized subcommittees improve both efficiency and decision quality.

### 4.8 Compensation and motivation

Most participants (95.4%) did not receive financial remuneration, with less experienced members more likely to be compensated. This finding consists of broader volunteerism literature, which indicates that initial engagement in voluntary roles is often financially incentivized, but sustained participation tends to be driven by intrinsic motivations.^15^

### 4.9 Communication and physician support

Open-ended feedback revealed strong desires for improved processes and better support for both CMs and patients. Many members were unaware of existing IMAO resources, highlighting the need for video training through HMC e-learning (eTaleem),^16^ collaboration with Hamad International Training Center (HITC),^17^ and enhanced subcommittee involvement, including timely feedback and clearer decision forms.

Patient-related challenges, particularly managing demanding or dissatisfied patients, were common. Similarly, Arrouf et al. identified unjustified patient requests, particularly requests for inappropriate sick leave certification and unnecessary antibiotic prescriptions, as the most frequent source of relational difficulties in patient–physician interactions. The study also highlighted the importance of adaptive communication strategies and maintaining professional boundaries when managing such situations.^18^

Actively involving physicians through a feedback system allows for satisfaction ratings and spontaneous suggestions. This structured feedback provides practical strategies, identifies recurring issues, and enables tailored interventions, enhancing physician-patient relationships and overall service quality. Pre-application education sessions can help inform patients and families about TA procedures and their rights.^19^ Providing patients with essential guidelines regarding the ethical standards of the TA committee decision-making, such as using official pathways (appeal requests) rather than challenging the decision-maker.^20^

### 4.10 Characteristics of an effective CM

Through our results, we established the “robot porter” model, representing an effective CM, an individual who is experienced within the corporate, though not necessarily within the committee, technologically competent, and selected to ensure equitable gender representation. This member prioritizes medical evidence above all other influences, maintains the human empathy required for patient-centered care, and works with appropriate compensation to acknowledge his contribution. Additionally, members must formally acknowledge their responsibilities by signing the committee charter and disclosing any potential conflicts of interest. This aligns with guidance from the Texas Healthcare Trustees (THT), which emphasizes selecting members based on expertise, clearly defining committee roles through a charter, and regularly evaluating operational efficiency.^20^

### 4.11 Transparency in IMAO Committee Decision-Making

In this study, transparency in TA committee decision-making refers to several key characteristics:

1. **Anonymity of patient requests**: Ensuring that CMs can review cases without patient identifiers reduces the potential for bias based on familiarity with the patient or prior interactions. This aligns with 52.9% of CMs supporting anonymized requests to facilitate impartial decisions.
2. **Clear documentation**: Decisions are accompanied by explicit reasoning, including clinical evidence, subcommittee input, and justification for approving or denying TA requests. This allows both stakeholders and patients to understand how conclusions are reached.
3. **Open communication**: Transparent communication includes timely updates between CMs, the IMAO team, and overseas facilities, ensuring decisions are informed, accurate, and reproducible.
4. **Accountability and COI management**: Transparency involves declaring potential conflicts of interest, reporting CMs’ activities and decisions through annual statistics publication.
5. **Consistency and standardization**: Structured processes, such as the involvement of specialized subcommittees and clear evaluation criteria, enhance predictability and fairness in decision-making, supporting a transparent workflow.

By defining transparency along these dimensions, the study highlights both procedural clarity and ethical accountability, demonstrating that transparent practices are integral to equitable, patient-centered decision-making and the protection of CMs.

**Figure 2.**
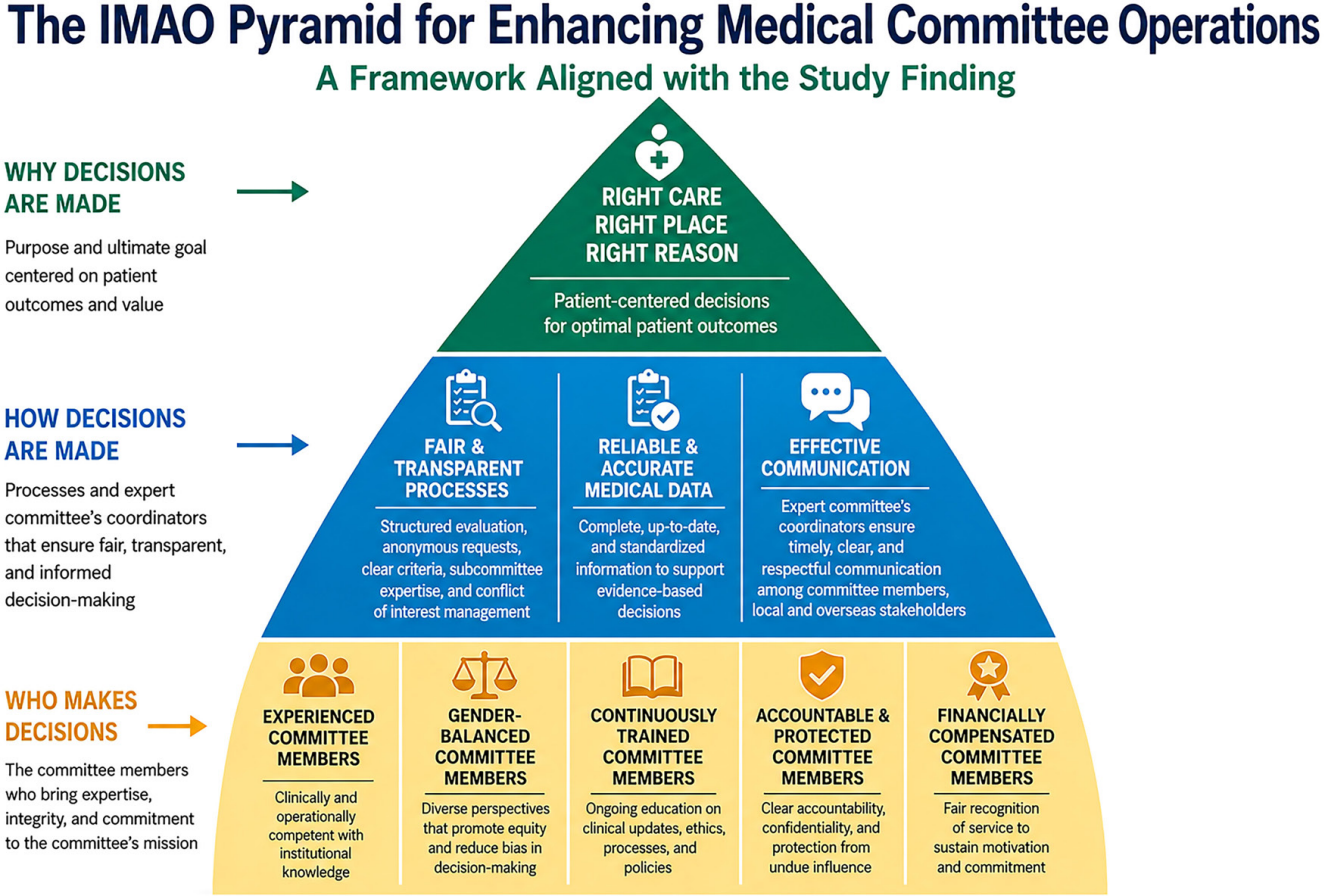
The hierarchical framework for committee operations.

To better conceptualize the factors influencing effective medical committee operations, we propose the IMAO pyramid framework (Figure 2). This model organizes key elements into three levels reflecting who makes decisions, how decisions are made, and why decisions are made. At the base are the experienced, gender-balanced, continuously trained, accountable, and compensated members responsible for decision-making. The middle layer emphasizes the importance of reliable and accurate medical data, fair and transparent processes, and effective communication led by trained coordinators. At the apex, the framework focuses on delivering the right care, in the right place, for the right reason, all guided by the goal of achieving the best possible patient outcomes. This pyramid, developed based on our study results, provides a strategic blueprint for strengthening medical committee effectiveness.

### 4.12 Study limitations 

Several limitations should be considered for this Phase I study. The survey achieved a response rate of 53.7%, which may not fully represent the perspectives of all CMs, respondents could have had stronger opinions or experiences than non-respondents. The study relied on self-reported data, which is subject to recall bias and social desirability bias, potentially affecting the accuracy of responses. Its cross-sectional design limits the ability to establish causal relationships, and longitudinal evaluation will be required to assess the impact of future interventions.

The study focused exclusively on the perspectives of CMs and did not include input from patients or their families, which could provide a more comprehensive understanding of the TA process. Additionally, the study was conducted within a single healthcare system, which may limit generalizability to other institutions or contexts. Finally, the proposed hierarchical framework has not yet been implemented; its practical effectiveness will be evaluated in Phase II through a follow-up survey assessing changes in satisfaction, decision-making quality, and operational performance.

## 5. CONCLUSION

The present study provides insights into the factors that influence effective medical committee decision-making within the IMAO Treatment Abroad system. As Phase I of a multi-phase quality improvement project, this work establishes a foundational understanding of the organizational, operational, and human factors that shape committee effectiveness. The findings demonstrate that committee effectiveness extends beyond clinical expertise alone and is fundamentally influenced by transparent governance, organizational experience, structured communication, and the supportive role of physician coordinators.

Institutional familiarity emerged as more influential than prolonged committee tenure in promoting member satisfaction and perceived effectiveness, highlighting the value of organizational knowledge and strategic member rotation in sustaining decision quality.

The study further illustrates that transparent and patient-centered decision-making relies on multiple interdependent elements, including accountable committee members, access to standardized and reliable medical data, multidisciplinary collaboration, and continuous professional development. Challenges, such as gender imbalance, workload intensity, and communication limitations, underscore the operational complexity of treatment abroad committees and suggest the need for clearer eligibility criteria, expanded subcommittee engagement, and strengthened administrative support.

The proposed IMAO Pyramid Framework integrates decision authority, decision processes, and decision purpose into a unified governance model. By explicitly linking structural, procedural, and ethical dimensions of decision-making, the framework offers a transferable approach for evaluating and strengthening committee governance in comparable healthcare settings. 

In Phase II of the study, the implementation and outcomes of the framework will be systematically assessed to explore its applicability and potential contribution to the broader literature on clinical decision-making and healthcare governance.

## ETHICAL APPROVAL 

The study was conducted in accordance with ethical guidelines for research involving human participants. Approval was obtained from the Corporate Quality and Patient Safety Research Committee (approval no. 09-2024) of Hamad Medical Corporation (HMC) before commencement. All participants were provided with detailed information about the study’s objectives, procedures, and their rights as participants.

## AUTHOR CONTRIBUTIONS

SA conceptualized the study, designed and implemented the survey, coordinated survey distribution and follow-up, performed data analysis and interpretation, developed the study framework, conducted the literature review, led all stages of the study as first investigator, drafted the original manuscript, and critically revised and finalized the manuscript for publication. AG contributed to advanced statistical analysis, data interpretation, preparation of figures/graphical presentations, and manuscript review. TM, RA, MA, MHK, and BV contributed to survey design and validation, data processing, and provided technical and methodological support. SA, YA, NA, KA, MQ, ZM, AE, and WG contributed to study quality assurance, survey development and design, participant response follow-up, and critical review and scientific editing of the manuscript. RS verified data integrity and accuracy and contributed to methodological refinement and correction. NG contributed to the survey design structure, layout development, and participated in manuscript review and editing. MA, AA, SG, MA, SA, AA, MB, EM, NK, JM, SS, AA, SA, AA, RA, NG, VP, MK, and SM contributed to survey follow-up and manuscript editing and review. All authors critically reviewed and approved the final manuscript for submission and publication.

## CONFLICT OF INTEREST STATEMENT

The authors declare that there are no conflicts of interest regarding the publication of this article.

## ACKNOWLEDGEMENTS

The authors would like to express their sincere gratitude to all IMAO committee members for their valuable participation, time, engagement, and thoughtful contributions to this study. Their active involvement, insights, and feedback were essential to the successful completion of the survey. We also extend our appreciation to the colleagues, reviewers, and subject-matter experts whose constructive comments and guidance.

## DISCLOSURE OF AI USE

Artificial intelligence-assisted tools were used exclusively for language editing and improvement of clarity and readability. These tools were not used to generate scientific content, analyze or interpret data, formulate conclusions, or make research decisions. All authors reviewed, verified, and approved the final manuscript and accepted full responsibility for its scientific accuracy and integrity.

## DATA AVAILABILITY STATEMENT

The data supporting the findings of this study are based on an anonymous survey. Key findings are presented in the article, but not all data are included due to the volume of information collected. Additional data are available from the corresponding author upon request.

## Figures and Tables

**Table 1.** Key insights and trends from the survey results (*N* = 87).

Category	Aspect	Result
Participants’ characteristics	Responders (out of 162)	87 (53.7%)
	HMC experience ≥9 years	75 (86.2%)
	IMAO experience ≥9 years	44 (50.6%)
	Gender (male/female)	63 (72.4%)/24 (27.6%)
Habits and organization	Prefer face-to-face	28 (32.2%)
	Prefer remote/hybrid	59 (67.8%)
	Prefer email	45 (51.7%)
	Satisfied with weekly meetings	81 (93.1%)
	Extra hours for the TA committee	48 (55.2%)
Interactions with IMAO team	Satisfied with collaboration	57 (65%)
	Satisfied with support	60 (68.9%)
Decision-making	Sub-committee absence considered as an obstacle for decision	20 (23%)
	Anonymous requests improve decision-making transparency	46 (52.9%)
	Decisions not influenced by third-party	71 (81.6%)
IMAO interface	Online interface challenges	16 (18.6%)
Compensation	No financial remuneration	83 (95.4%)

**Table 2a.** Differences in perceptions of the IMAO team by committee members’ experience within HMC.

Grouping variable	Dependent Variable	*N* (%): Less experienced/more experienced	Mean Rank: Less experienced/more experienced	Mann-Whitney *U*	*Z*	*P* value
Experience within the organization (HMC) (<9 years / >9 years)	Relationship with the IMAO coordinators	12 (13.8%)/75 (86.2%)	29.42/46.33	275,000	−2.258	0.024
	Satisfaction with the IMAO physician’s medical summary	12 (13.8%)/75 (86.2%)	27.38/46.66	250,500	−2.556	0.011
	Help provided by the IMAO team when you request assistance	12 (13.8%)/75 (86.2%)	30.33/46.19	286,000	−2.108	0.035

**Table 2b.** Differences in perceptions of the IMAO team by committee members’ experience within the IMAO committee.

Grouping variable	Dependent Variable	*N* (%): Less experienced/more experienced	Mean Rank: Less experienced/more experienced	Mann-Whitney *U*	*Z*	*P* value
Experience as an IMAO committee member (<9 years / >9 years)	Relationship with IMAO coordinators	43 (49.5%) / 44 (50.6%)	44.69 / 43.33	916,500	−0.263	0.793
	Satisfaction with the IMAO physician medical summary	43 (49.5%) / 44 (50.6%)	42.06 / 45.90	862,500	−0.738	0.461
	Help provided by the IMAO team when you request assistance	43 (49.5%) / 44 (50.6%)	45.80 / 42.24	868,500	−0.687	0.492

**Table 3.** Committee members’ suggestions and feedback.

Key theme	Treatment abroad committee members’ feedback
Interface	“Provide guides, tutorials, and FAQs to help.” “Less steps to login.” “To create an application to access from the mobile.”
Communication	“More specific questions for the members, rather than approved or not.” “Add more methods of communication.”
Patient medical information	“Referring to reports from abroad: ‘…a lot of irrelevant information.’” “On many occasions, it is not clear why the patient needs to go abroad.” “Gaps in medical information from the patient’s treatment center abroad.”
Compensation for extra work	“Why are members not compensated like other committees?” “Compensation is recommended for tasks often done after clinical hours.”
Increased transparency	“Statistics should be published, numbers of approvals, rejections, costs.” “COI statement of each member should be in files.”
Patient education	“Sometimes the patient refuses the treatment locally, sending him will save his life.” “Protect committee members from aggressive applicants.” “We should decrease the number of outgoings for treatment abroad.”
Relationship with the IMAO team	“Thank you very much for generating this survey.” “I am satisfied; they are doing a great job.”

## APPENDIX 1

### IMAO Committee Members Questionnaire

Please complete the following questionnaire regarding your experience and participation as an IMAO committee member.

**1. Participant Characteristics**

- How long have you been working in the organization (HMC/Sidra)?
  ⎕ ≥ 9 years ⎕ < 9 years

- How long have you been an IMAO committee member?
  ⎕ ≥ 9 years ⎕ < 9 years

- Please specify your gender:
  ⎕ Male ⎕ Female

**2. Participant Habits**

- What means of communication do you use to communicate with the IMAO team?
  ⎕ Orally over the phone
  ⎕ Email
  ⎕ Text and WhatsApp messages

- Do you nominate another member when you need to be away from the committee?
  ⎕ Yes ⎕ No

- In addition to your prescribed working hours, do you dedicate extra hours to fulfill your duties as a committee member?
  ⎕Yes ⎕ No

**3. Committee Organization**

- Are you satisfied with the frequency of the committee meetings (weekly)?
  ⎕ Yes
  ⎕ No, please suggest an alternative: ____________________

- Are you satisfied with the scheduled day of the committee meeting (Tuesday)?
  ⎕ Yes
  ⎕ No, please suggest an alternative: ____________________

- What type of participation in committee meetings do you prefer?
  ⎕ Face-to-face
  ⎕ Remotely
  ⎕ Hybrid

**4. Committee Decision-Making**

- Is the absence of a sub-committee decision considered an obstacle in your decision-making?
  ⎕ Yes ⎕ No ⎕ Not related

- Is it challenging to decide on non-medical requests (e.g., extra escorts or ticket upgrades)?
  ⎕ Yes ⎕ No

- Has your decision ever been influenced by the patient’s name?
  ⎕ Yes ⎕ No

- Do you think anonymous patient requests could improve transparency in decision-making?
  ⎕ Yes ⎕ No

- Has your decision-making ever been influenced by a third party (e.g., recommendations from a high-ranking individual)?
  ⎕ Yes ⎕ No

**5. Collaboration with the IMAO Team**

- How would you rate your relationship with the IMAO physician coordinators?
  ⎕ Very dissatisfied ⎕ Dissatisfied ⎕ Neutral ⎕ Satisfied ⎕ Very satisfied

- Are you satisfied with the quality of the IMAO physicians medical summaries?
  ⎕ Very dissatisfied ⎕ Dissatisfied ⎕ Neutral ⎕ Satisfied ⎕ Very satisfied

- How do you rate the support provided by the IMAO team when assistance is requested?
  ⎕ Very dissatisfied ⎕ Dissatisfied ⎕ Neutral ⎕ Satisfied ⎕ Very satisfied

**6. Compensation**

- Are you receiving any in-kind benefits from your organization for being a committee member?
  ⎕ Yes ⎕ No
  If yes, please specify: ____________________

- Are you receiving overtime financial compensation for being a committee member?
  ⎕ Yes ⎕ No

- Are you receiving any financial remuneration from your organization for being a committee member?
  ⎕ Yes ⎕ No

**7. IMAO Committee Interface**

- Are you facing challenges accessing the IMAO committee online interface?
  ⎕ Yes ⎕ No

- How can the IMAO committee interface be improved?
  ______________________________________________________

- **General comments and suggestions**:
  ______________________________________________________
